# Alterations of color vision and central visual field in patients with Vogt−Koyanagi−Harada syndrome

**DOI:** 10.1007/s12348-011-0055-5

**Published:** 2012-02-02

**Authors:** Peizeng Yang, Min Sun, Xiaoli Liu, Hongyan Zhou, Wang Fang, Li Wang, Aize Kijlstra

**Affiliations:** 1 The First Affiliated Hospital of Chongqing Medical University, Chongqing Key Laboratory of Ophthalmology, Youyi Road 1, Chongqing, 400016 People’s Republic of China; 2 Zhongshan Ophthalmic Center, Sun Yat-sen University, Guangzhou, People’s Republic of China; 3 Eye Research Institute Maastricht, Department of Ophthalmology, University Hospital Maastricht, Maastricht, the Netherlands

**Keywords:** Vogt–Koyanagi–Harada syndrome, Color vision, Visual field

## Abstract

**Purpose:**

To investigate the changes of color vision and central visual field in a cohort of patients with Vogt–Koyanagi–Harada (VKH) syndrome.

**Methods:**

Sixteen VKH patients (32 eyes) were enrolled in this study. All the patients were treated with immunosuppressive agents. The best visual acuity, visual field testing and color vision testing were available from the records in all these patients at different time points, i.e. before treatment and 1 month (±7 days), 3 months (±15 days), 6 months (±20 days) and 12 months (±30 days) after treatment.

**Results:**

All patients showed active intraocular inflammation at their first visit. A decreased visual acuity, abnormality of color vision and abnormal visual field were observed at presentation. Visual acuity and color vision rapidly improved at 1 and 3 months and gradually improved thereafter. Visual field defects significantly improved at 6 months and gradually improved thereafter. However, visual field defects were still observed in 27.5% of the tested patients following a 12-month treatment. Color vision returned to the normal level only in about one-third of these patients at this time point.

**Conclusions:**

Visual function was severely impaired in VKH patients with active uveitis but rapidly improved following immunosuppressive therapy. Visual fields are much more severely affected by the disease than visual acuity and its improvement lagged behind that of visual acuity and color vision.

## Introduction

Vogt–Koyanagi–Harada (VKH) syndrome has generally been considered as an autoimmune disease against melanocytes. It is characterized by choroiditis at the onset of disease and a granulomatous uveitis at recurrence. A variety of extraocular manifestations are associated with this syndrome [1–4]. It is more prevalent in certain pigmented races, such as Asians and Native Americans [1–4]. Visual prognosis is generally favorable when prompt diagnosis was made and appropriate immunosuppressive treatment was installed [2, 5, 6].

The central visual acuity is considered to be an important measurement of visual function and is an important indicator of visual outcome in patients with VKH syndrome [2, 5]. However, in our clinical experience, some patients with good or even normal central visual acuity still complain of visual disturbances such as visual field defects or abnormal color vision. In a recent study, we have revealed that certain VKH patients with normal visual acuity have overt abnormalities when tested by multifocal electroretinography [7]. Therefore, visual acuity measurement alone is not a sufficient parameter to accurately evaluate the retinal, especially the macular function. Central visual field and color vision have been shown to be important parameters for visual function [8]. In this study, we evaluated the alterations of color vision and central visual field in patients with VKH syndrome to better understand the effects of disease and treatment on visual function.

## Material and methods

This retrospective study included patients diagnosed as VKH syndrome between January 1, 2006 and March 20, 2008. The diagnosis of VKH was based on detailed history and clinical examinations described in an earlier study [5]. All patients included in this study met the revised criteria for VKH syndrome by the International Nomenclature Committee [9]. A history or the presence of exudative retinal detachment and pigmentary changes involving the macular area was inquired or observed in all of the patients at first visit or follow-up visit in our hospital. Hereditary color vision defects were carefully inquired in each patient and no color vision defects were reported by any of them. Patients with conditions which might alter the color vision and visual field values, such as a history of glaucoma, presence of increased intraocular pressure, overt media opacity, posterior synechiae, a history of previous ocular surgery, or diabetes were excluded from the study.

The patients were followed up and examined using Snellen charts at each visit following treatment. Visual field testing and color vision testing were available from the records in all these patients at different time points, i.e. before treatment and 1 month (±7 days), 3 months (±15 days), 6 months (±20 days) and 12 months (±30 days) after treatment. These tests were performed in the routine facilities of the institute and the technicians administering the test were unaware of the diagnosis of the patients. A thorough ophthalmological examination, including slit-lamp biomicroscopy and ophthalmoscopy through a dilated pupil, was performed in all patients. For the purpose of statistical analysis, Snellen chart visual acuity was converted to the logarithm of the minimal angle of resolution (log MAR) value [10]. Visual fields were tested with the Humphrey Field Analyzer Program 30–2 according to the Humphrey perimetry manual. The efficiency of fixation of the subjects was constantly monitored by an experienced perimetrist. The mean deviation (MD, in decibels) and corresponding *P* value of each field analysis was noted. The Farnsworth-Munsell (FM) 100-hue test under standard illuminant C conditions was performed to detect color vision defects [8].

For purpose of statistical analysis, normal visual acuity was defined as best-corrected visual acuity (BCVA) with equal to or better than 0.8 in this study. Visual fields with false-positive responses of more than 15%, false-negative responses, or a rate of fixation loss of more than 20% were not included in this analysis. In this study, we used MD and *P* value to evaluate the visual field abnormality. MD value reflected the extensive damage of the retina. The more severe the retina damage was, the lower value of MD. Each *P* value corresponds to a MD value. The *P* value indicates the probability of that MD being found in the normal population. Color vision scores were determined using the FM 100 testing which was performed in a dark room with test materials viewed under standard illuminant conditions. Subjects used near vision correction, if needed, and were given 2 min to complete each of the four test boxes. The approximate distance from the subjects’ eyes to the test materials was 30 to 50 cm. Total error score was calculated according to the manufacturer’s recommendations, and the square root of the total error score was used in analyses. A high value reflects a defect of color vision. For normal color vision values, we chose data from a population of healthy Chinese individuals previously reported by Huang and associates [11] who also measured the color vision with the FM 100 test.

The study followed the tenets of the World Medical Association Declaration of Helsinki. Informed consent was obtained from all patients prior to these aforementioned examinations and treatment with immunosuppressive agents.

The data were collected from the patients and were subsequently entered into a computer-based data bank. Statistical analysis was performed using SAS for windows XP. The ANOVA was used to test the significance of the mean values before treatment and the values at 1, 3, 6 and 12 months after treatment. A *P* value less than or equal to 0.05 was considered statistically significant.

## Results

This retrospective study included 32 eyes of 16 patients. There were nine males and seven females in the study patients. The mean age was 33.2 ± 9.6 years, ranged from 21 to 50 years old. All the patients with VKH syndrome had an active intraocular inflammation at their first visit and were treated with corticosteroids in combination with other immunosuppressive agents such as cyclosporine or chlorambucil. The ocular findings included diffuse choroiditis, exudative retinal detachment, optic disk edema or overt granulomatous anterior uveitis in association with ‘sun-set glow’ fundus and nummular chorioretinal depigmented scars.

**Fig. 1:**
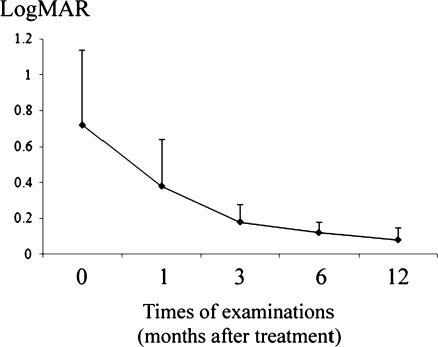
Time course of best-corrected visual acuity (in terms of *LogMAR*) following immunotherapy in VKH eyes, the *plots* mean the average of the test, the *error bars* mean standard deviation: SD

The BCVA ranged from 20/250 to 20/25 (median BCVA = 20/71) on Snellen charts, and the mean logMAR value was 0.72 (SD = 0.42) before treatment. One month after treatment, the BCVA ranged from 20/200 to 20/16 (median BCVA = 20/33), and the mean logMAR value was 0.38 (SD = 0.26), significantly lower than that before treatment (*P* = 0.026). Three months after treatment, the BCVA ranged from 20/80 to 20/15 (median BCVA = 20/28), and the mean logMAR value was 0.18 (SD = 0.10; *P* = 0.012). The BCVA continued to improve at 6 months (median BCVA = 20/24, mean logMAR = 0.12 ± 0.06, *P* < 0.001) and 12 months (median BCVA = 20/21, mean logMAR = 0.08 ± 0.07, *P* < 0.001; Fig. 1). At the final follow-up, 81.3% of the eyes had a BCVA of ≥20/25 and only 6.3% of the eyes had a BCVA below 20/40.

**Fig. 2:**
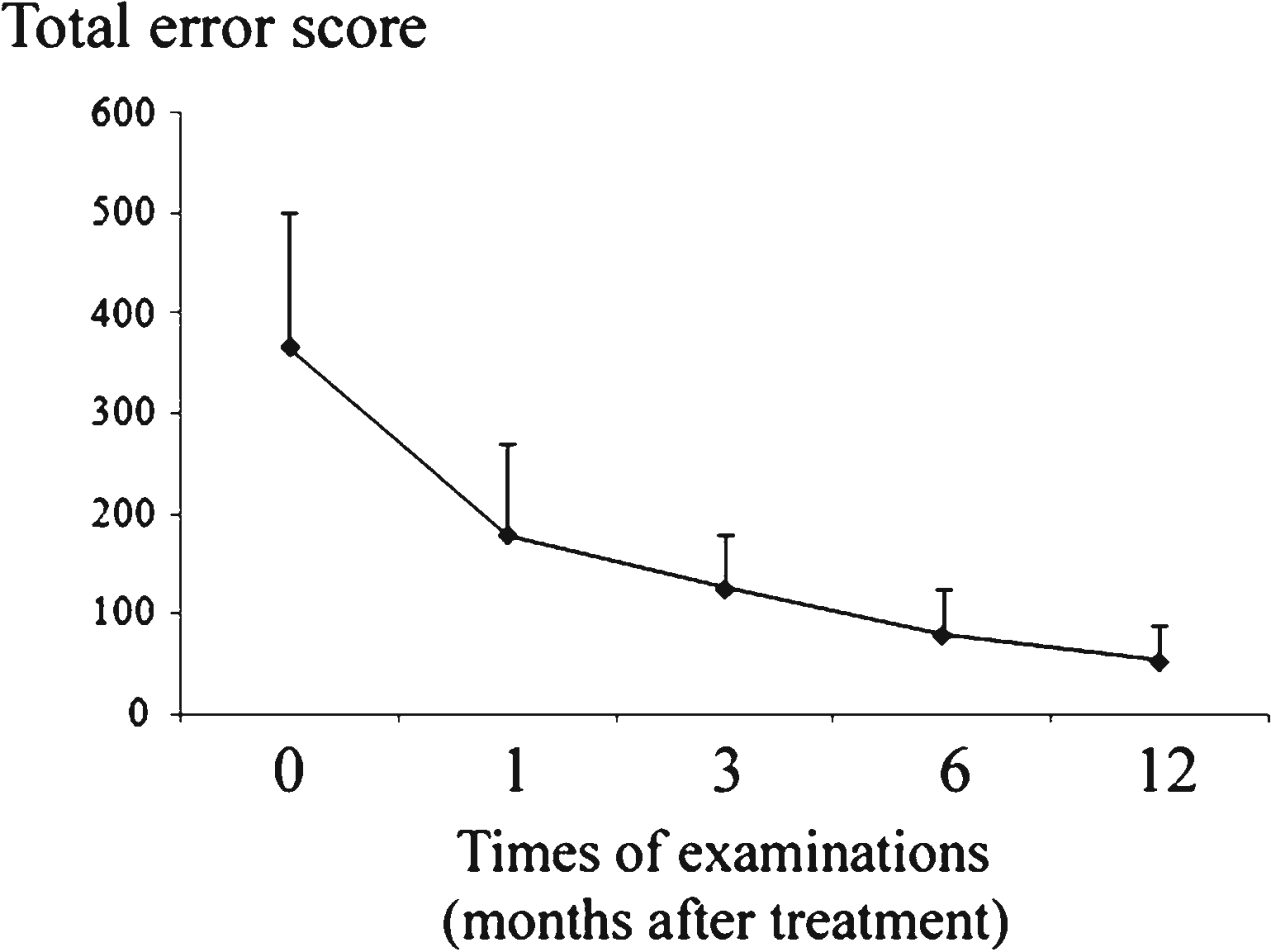
Time course of color vision changes (in terms of total error score, TES) following immunotherapy in VKH eyes, the *plots* mean the average of the test, the *error bars* mean standard deviation: SD

FM 100-hue testing showed that the mean total error score (TES) in these patients was 365 (SD = 135) before treatment. One month after treatment, the mean TES decreased to 178 (SD = 91), showing a significant improvement (*P* = 0.029). The mean TES was further improved at 3 months (124 ± 56, *P* < 0.001), 6 months (78 ± 46, *P* < 0.001) and 12 months (52 ± 36, *P* < 0.001; Fig. 2). However, the mean TES was still abnormal in 65.6% of the tested patients at 12 months following treatment.

**Fig. 3:**
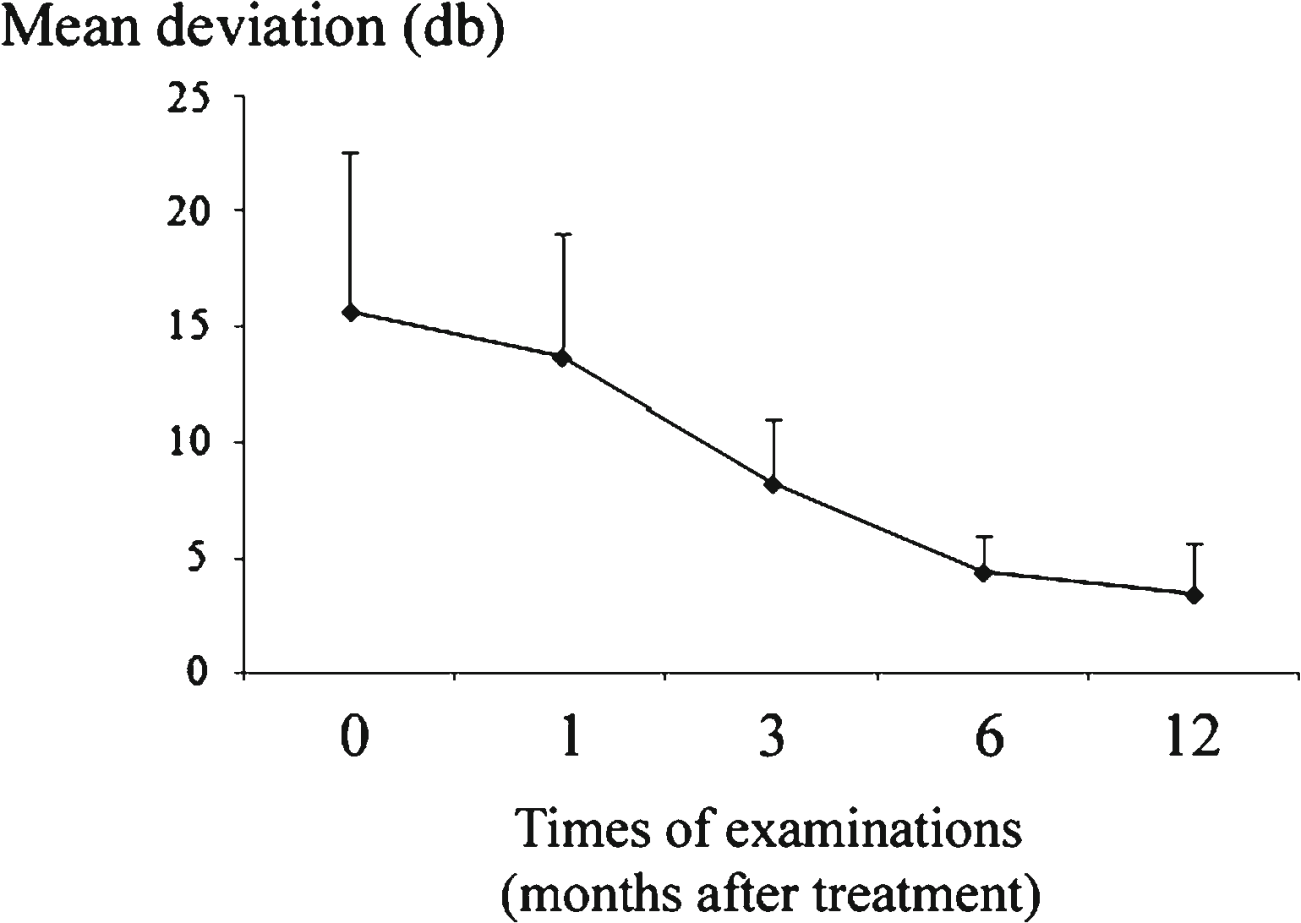
Time course of visual field changes (in terms of mean deviation, MD) following immunotherapy in VKH eyes, the *plots* mean the average of the test, the *error bars* mean standard deviation: SD

The MD measured with the Humphrey Field Analyzer ranged from 6.82 to 26.02 db, with a mean value of 15.62 db (SD = 6.98) in the VKH patients before treatment. One month after treatment, the MD value ranged from 6.20 to 17.59 db, with a mean value of 13.64 (SD = 5.38; *P* = 0.102). Three months later, the mean MD value was 8.20 db (SD = 2.80; *P* = 0.087).The mean MD value was significantly improved at 6 months (4.34 ± 1.58, *P* = 0.026) and 12 months (3.37 ± 1.21, *P* = 0.013; Fig 3). At 12 months following treatment, the MD value returned to normal levels in 72.5% of these patients. The mean *P* value was lower than 1.52% in the VKH patients before treatment. One and 3 months after treatment, the mean *P* value was lower than 0.72% (*P* = 0.448) and 0.76% (*P* = 0.426), respectively. At 6 months after treatment, the mean *P* value was lower than 2.32%, which was slightly higher than that before treatment although there was no statistical difference (*P* = 0.325). After 12-month treatment, the mean *P* value was lower than 3.86%, which was significantly higher than that before treatment (*P* = 0.032).

## Discussion

In this study, we evaluated the visual function of VKH patients with active uveitis before and after treatment with immunosuppressive agents using visual acuity, color vision and visual field. Our results showed that the patients displayed striking abnormalities in these three parameters before treatment, reflecting a severely impaired visual function in active VKH syndrome. An improvement of visual acuity and color vision was rapidly achieved at 1 and 3 months, followed by a gradual recovery at 6 and 12 months after treatment. For the visual field results, a marked amelioration was observed at 6 months and thereafter following treatment. These results show that there is a substantial recovery of visual function in VKH patients following appropriate immunosuppressive treatment. Among the three tested parameters, recovery of visual acuity and color vision preceded an improvement of visual field.

Various studies have shown that visual acuity at presentation is an important prognostic indicator of final visual acuity in patients with VKH syndrome [2, 5]. Approximately 60–80% of patients could achieve a visual acuity of 20/40 or better [2, 5, 6]. In our patients, we found that more than 90% had a visual acuity of 20/40 or better at the final follow-up. Furthermore, a rapid decrease of the logMAR value was observed at 1 and 3 months following treatment, indicating a prompt recovery of visual acuity in VKH patients following appropriate treatment. This result is generally consistent with that reported by Rubsamen et al. [12].

A number of tests have been designed for color vision screening and grading. The Farnsworth–Munsell 100-hue test is designed to measure hue discrimination ability at a constant value and chroma and is considered an excellent choice in evaluating color vision defects [13].To rule out other causes of color vision defects, we excluded patients with conditions which could influence the color vision. Interestingly, we found that abnormal color vision was present in all the examined VKH patients with active uveitis. As color vision generally reflects the function of retinal cone cells [14], it is reasonable to presume that these cells were severely involved during VKH syndrome. Our results are in agreement with previous reports in which abnormal color vision is common in retinochoroidal or optic nerve disorders, such as diabetic retinopathy, tilted disc syndrome, optic neuritis and primary open angle glaucoma [15–18]. Abnormal color vision has also been reported in birdshot choroiretinopathy [19]. It is unknown whether abnormal color vision is a universal finding of uveitis involving the choroid and retina. Further study is required to clarify this issue. Similar to visual acuity, color vision rapidly improved after immunosuppressive treatment.

Visual field is another important parameter to evaluate visual function. Our study showed severe visual field abnormalities in VKH patients before treatment as evidenced by severely decreased MD value and *P* value. Visual field has been shown to reflect the function of the retina, choroid and optic nerve [20]. There are numerous field abnormalities observed in ocular fundus diseases. The impaired visual field is generally in accordance with the inflammatory damage to the choroid and its adjacent tissues observed in this syndrome. Clinically, this disease is characterized by choroiditis, chorioretinitis and neuroretinitis at its first attack [2, 5]. Pathologically, marked losses of melanocytes and infiltration of lymphocytes in the choroid, and destruction or hyperplasia of retinal pigment epithelium (RPE) have been described in VKH syndrome [21]. In addition, fundus fluorescein angiography [22], indocyanine green angiography [23] and optical coherence tomography [24] also showed a variety of changes in the choroid, retina and RPE. Similar to visual acuity and color vision, central visual field also significantly improved following appropriate treatment. However, it is unexpected that the recovery of both the MD value and *P* value lagged behind that of visual acuity. The visual field results are in general accordance with those presented by Gordon et al. [20] in birdshot choroiretinopathy. They also found that the visual field test is independent of other measures of visual dysfunction and that visual field recovery lagged behind that of visual acuity. These observations may suggest important features concerning the pathology of both diseases. More studies are needed to clarify this issue. An unsynchronized recovery of visual acuity, central visual field and color vision in patients with VKH syndrome suggests that sub-clinical abnormal macular function is still present in certain patients although the intraocular inflammation is completely controlled.

In summary, our studies showed that a severely decreased visual acuity was associated with abnormal color vision and visual field defects in patients with active VKH syndrome. Following appropriate immunosuppressive treatment, improvement of visual acuity and color vision was faster than that of the visual field. Both color vision and visual field parameters may provide additional quantifiable measurements of visual dysfunction in patients with VKH syndrome. However, it is not yet known whether visual field or color vision at baseline is a better predictor of final visual acuity. Further studies are needed to clarify this issue.
